# Validation of the Brazilian-Portuguese Version of the Clinician Administered Post Traumatic Stress Disorder Scale-5

**DOI:** 10.3389/fpsyt.2021.614735

**Published:** 2021-06-22

**Authors:** Thauana Torres Oliveira-Watanabe, Luis Francisco Ramos-Lima, Cecilia Zylberstajn, Vinicius Calsavara, Bruno Messina Coimbra, Mariana Rangel Maciel, Lucia Helena Machado Freitas, Marcelo Feijo Mello, Andrea Feijo Mello

**Affiliations:** ^1^Post-Graduate Program in Medical Psychology and Psychiatry, Department of Psychiatry, Federal University of São Paulo, São Paulo, Brazil; ^2^Program for Research and Care on Violence (PROVE) and Post Traumatic Stress Disorder, Federal University of São Paulo, São Paulo, Brazil; ^3^Psychological Trauma Research and Treatment Program, Post Traumatic Stress Disorder, Clinical Hospital of Porto Alegre, Porto Alegre, Brazil; ^4^Post-Graduate Program in Psychiatry and Behavioral Sciences, Federal University at Rio Grande Do Sul, Porto Alegre, Brazil

**Keywords:** post-traumatic stress disorder, assessment, instrument validation, clinician-administered PTSD scale, psychometric evaluation validity/reliability

## Abstract

**Objectives:** The aim of this study was to validate CAPS-5 for the Brazilian-Portuguese language on a sample of 128 individuals from two centers (from the cities of São Paulo and Porto Alegre) who have been recently exposed to a traumatic event.

**Methods:** We performed a reliability analysis between interviewers (with a subset of 32 individuals), an internal consistency analysis, and a confirmatory factorial analysis for the validation study.

**Results:** The inter-rater reliability of the total PTSD symptom severity score was high [intraclass correlation coefficient =0.994, 95% CI (0.987–0.997), *p* < 0.001]. Cohen's Kappa for individual items ranged between 0.759 and 1. Cronbach's alpha coefficients indicated high internal consistency for the CAPS-5 full scale (α = 0.826) and an acceptable level of internal consistency for the four symptom clusters. The confirmatory factorial analysis for the 20-item original CAPS-5 did not fit the data well. A 15-item model with better results was then established by excluding the following CAPS-5 items: dissociative amnesia, recklessness, distorted cognitions, irritability, and hypervigilance.

**Conclusion:** Despite the limitation of the predominance of female victims, and the high number of sexually assaulted women in our sample, the model with only 15 items provided a good fit to the data with high internal consistency (α = 0.835).

## Introduction

Post-traumatic stress disorder (PTSD) is a severe psychiatric condition developed after exposure to a traumatic event (1). Since 1980, when PTSD was first included in the third version of the Diagnostic and Statistical Manual of Mental Disorders (DSM-III), the definition has been changed and updated in the versions that followed (2). A traumatic event is required for the diagnosis of PTSD, and this has been highlighted in DSM-5, as PTSD is no longer classified among Anxiety Disorders, but in a specific category of Trauma and Stressor-Related Disorders (3). Other changes to the classification of PTSD have restricted what qualifies as a traumatic event and have split the symptoms into four clusters: reexperience, avoidance, negative thoughts and cognitions, and hyperarousal.

Due to the burden of traumatic events, the World Health Survey Consortium conducted a study, which demonstrated that 70.4% of the respondents of all countries had experienced at least one traumatic event in their lifetime (4). In Brazil, this number is even higher: ~80% of the Brazilian population has experienced a traumatic event, especially related to urban violence (5). This estimate raises great concern to the Brazilian public health system; an epidemiological study demonstrated that 10.2% of the trauma-exposed population in São Paulo and 8.7% in Rio de Janeiro develop PTSD (2). PTSD can cause a poorer quality of life, which consequently burdens health and social public services (3).

Due to the high prevalence of PTSD in Brazil, its proper assessment and diagnosis is crucial. Currently, the instrument recognized as the gold standard for evaluating PTSD is the clinician-administered PTSD scale (CAPS-5), a structured diagnostic interview to be applied by clinicians. In Brazil, although ICD-10 is the most used classification system regarding general medical diagnosis, concerning psychiatric diagnosis and research purposes DSM-5 is more used than ICD-10. The original scale (6), developed in English, has been last updated to match the DSM-5 PTSD diagnostic criteria (7). The scale has demonstrated high internal consistency, inter-rater reliability, and test-retest reliability. CAPS-5 also demonstrated good diagnostic correspondence with CAPS-IV (7) and has already been validated in other languages, such as Dutch and German (8, 9).

Adapting an instrument to a specific language has great significance, not only because of the language itself, but also because of the impact that differences in culture, beliefs, and behaviors have on understanding mental health (10). CAPS-5 was validated in the United States on predominantly male veterans (7), a very specific population that greatly differs from the PTSD population in the public health system of Brazil (11).

Brazilian epidemiological studies have revealed that women tend to be more often diagnosed with PTSD than men (12, 13). Ribeiro et al. (2) evaluated the conditional risk of developing PTSD following a traumatic event and found a three-fold increased risk of developing the disorder in females compared to that in males [15.9% in females (95% CI 14.2–17.6) vs. 5.1% in males (95% CI 4.0–6.2)]. These findings highlight the importance of adapting the original version of CAPS-5 to the Brazilian-Portuguese language and validating the instrument to better conduct research in Brazil.

The aim of this study was to validate CAPS-5 for the Brazilian-Portuguese language. Previously, our research team performed a cross-cultural adaptation process with a formal and structured methodology to ensure conceptual, semantic, and operational equivalence (14). In order to complete the validation process, we performed a reliability analysis between interviewers, evaluated internal consistency, and conducted a confirmatory factorial analysis (CFA). We hypothesized that the instrument would exhibit good inter-rater reliability and internal consistency, based on psychometric measurements obtained in previous studies performed in other countries (7–9).

## Methods

### The CAPS-5 Instrument

The scale assesses the diagnostic criteria based on DSM-5 and the intensity of the PTSD symptoms; CAPS-5 is considered the gold standard in the diagnosis of PTSD. It has 30 questions, 20 of which correspond to each DSM-5 diagnostic criterion. The first question refers to the existence of a traumatic experience (Criteria A); the original scale recommends another instrument to evaluate the occurrence of traumatic events, usually the Life Events Checklist (LEC-5) (15). The LEC-5 is a self-report measure designed to recognize potentially traumatic events in a respondent's lifetime. We also used LEC-5 to evaluate Criteria A, adapted to Brazilian-Portuguese in a previous study (16).

The 20 symptom-related questions were divided into four groups: intrusion symptoms (five items, Criterion B), avoidance questions (two items, Criterion C), negative alterations in cognition and mood (seven items, Criterion D), and hyperarousal (six items, Criterion E). Concerning other DSM-5 criteria, one question refers to how long the identified traumatic event lasted (Criteria F) and three questions to the impact of the disturbance on functionality (Criteria G). Three final questions regarding the interviewer's impression on the patient are presented, along with two questions regarding the presence of dissociative symptoms.

To evaluate frequency and intensity, which is separately assessed, the scale rated intensity as *minimal, clearly present, pronounced*, and *extreme*, and frequency is recorded as reported by the respondent as the number of times the symptom is present. After that, severity can be rated: 0 = *absent*, 1 = *mild/subthreshold*, 2 = *moderate/threshold*, 3 = *severe/markedly elevated*, and 4 = *extreme/incapacitating*. The symptom is consider present if its severity rating is 2 or higher (7).

### Cross-Cultural Adaptation

The cross-cultural adaptation process was performed within the Program for Research and Care on Violence and PTSD (PROVE) at the Department of Psychiatry of The Federal University of São Paulo (UNIFESP) according to the model proposed by Reichenheim and Moraes (10). This process included the following steps: translation from English to Portuguese, a back-translation to the original language, a revision by an expert team, a pilot study using the adapted version, and an equivalence evaluation with the original version. This adaptation was published previously (14).

### Participants

This study was conducted in two centers: at UNIFESP and at the Clinical Hospital of Porto Alegre (HCPA), which belongs to the Federal University of Rio Grande do Sul (UFRGS). The participants spontaneously sought psychiatric assistance after experiencing a traumatic event and were enrolled from the screening process of both centers, except for sample 1 described below, whose patients were addressed to UNIFESP after the first evaluation at a gynecological center, specialized to attend sexual abuse. In order to establish PTSD diagnosis, an experienced psychiatrist in attending PTSD patients performed a clinical evaluation in every patient during triage. All the participants who fulfilled the PTSD diagnostic criteria by DSM 5 after clinical assessment were invited to and agreed to participate in the study. Other inclusion criteria were to be able to understand the informed consent term (ICT), to be able to read and sign the ICT and being older than 18 years-old. Exclusion criteria were having other diagnosis besides PTSD (comorbid depression and anxiety were not excluded), and significant intellectual deficit.

The complete sample was composed of three groups of patients:

The first group (**Sample 1**) consisted of sexually assaulted women included in a randomized clinical trial, which is part of a thematic project sponsored by Fundação de Amparo a Pesquisa do Estado de São Paulo (FAPESP), conducted at PROVE-UNIFESP (17). The patients were enrolled between January 2016 and March 2019. The adapted CAPS-5 was applied during the screening process to select the participants for the study, together with other instruments concerning the thematic project. The scale was administrated by two trained professionals (one psychiatrist and one psychologist). All patients experienced the trauma up to 6 months before the assessment.

The second group (**Sample 2)** was enrolled from the screening process of PROVE outpatient's service. The participants spontaneously sought for psychiatric assistance after experiencing a traumatic event. The screening process was conducted by two professionals (one psychiatrist and one psychologist), who also applied the adapted version of CAPS-5. The patients were enrolled between March 2018 and February 2019.

The third group (**Sample 3)** was enrolled at the Psychological Trauma Research and Treatment Program (NET-Trauma) outpatient service from HCPA-UFRGS. The screening process was similar to the PROVE outpatient center; patients suffering different types of trauma agreed to participate in the study. They were assessed between August 2018 and February 2019. A summary of the three samples is presented in Table 1.

**Table 1:** Sociodemographic data from three samples (*n* = 128).

**Variables**	**Sample 1**	**Sample 2**	**Sample 3**	**All**	***P-*value**
	**(*n* = 80)**	**(*n* = 17)**	**(*n* = 31)**		
Age (mean, sd)	25.5 (6.7)	37.8 (11.1)	35.9 (16.2)	29.7 (11.6)	<0.001^a^
Female gender	80 (100%)	14 (82.4%)	26 (83.9%)	120 (93.8%)	0.001^a^
**Ethnicity**					
Caucasian	32 (40.0%)	7 (43.8%)	19 (61.3%)	58 (45.7%)	<0.001^a^
African-American	46 (57.5%)	8 (50.0%)	5 (16.1%)	59 (46.5%)	
Asiatic-American	2 (2.5%)	1 (6.3%)	7 (22.6%)	10 (7.9%)	
**Marital status**					
Single	55 (68.8%)	9 (60.0%)	16 (51.6%)	80 (63.5%)	0.006^a^
Married/engaged	23 (28.7%)	4 (26.7%)	7 (22.6%)	34 (27.0%)	
Divorced/widower	2 (2.5%)	2 (13.3%)	8 (25.8%)	12 (9.5%)	
**Educational level**					
<4 years	0 (0%)	0 (0%)	2 (6.5%)	2 (1.6%)	0.001^a^
4–12 years	39 (48.8%)	2 (16.7%)	22 (71.0%)	63 (51.2%)	
>12 years	41 (51.2%)	10 (83.3%)	7 (22.6%)	58 (47.2%)	
**Employment status**					
Employed	63 (78.8%)	8 (66.7%)	15 (48.4%)	86 (69.9%)	0.011^a^
Unemployed/retired	13 (16.3%)	4 (33.3%)	10 (32.3%)	27 (22.0%)	
Health licensed	4 (5.0%)	0 (0%)	6 (19.4%)	10 (8.1%)	
Religious	55 (68.8%)	7 (53.8%)	21 (67.7%)	83 (66.9%)	0.567
Caps severity score (IQR)	41.5 (35.0–49.0)	40.0 (34.5–46.0)	42.0 (30.0–52.0)	41.5 (33.2–49.0)	0.955
# symptoms	15.0 (13.0–17.0)	15.0 (13.0–16.0)	15.0 (11.0–17.0)	15.0 (12.2–17.0)	0.529

a *p < 0.05*.

*sd, standard deviation; IQR, Inter-quartile Range*.

All the professionals who applied the instrument were trained for CAPS-5 use. In every case, the LEC-5 scale was applied to ensure that the DSM-5 Criteria A for PTSD was fulfilled. This study was conducted with approval from the Ethics Committees of both UNIFESP and HCPA-UFRGS. All participants signed the informed consent form.

### Data Analysis

#### Reliability Between Interviewers

We compared the results of two independent interviewers (one psychiatrist and one psychologist), who had administered CAPS-5 to the same participants. All ratings were performed by the same two raters between different groups. For the reliability evaluation between interviewers, 32 participants were selected from Sample 1 (*n* = 15 participants) and 2 (*n* = 17 participants). We calculated the Cohen's kappa coefficient, considering a confidence interval of 95% and the 20 items of the scale as ordinal variables. The kappa coefficient varies between 1 and −1, which indicates complete agreement or complete disagreement, and a value of 0 indicates a random result (18). This coefficient was applied to all of the 20 items of the scale corresponding to DSM-5 symptoms. We also used the intraclass correlation coefficient to evaluate the total PTSD severity score (19).

#### Internal Consistency

We combined the three samples in order to obtain the minimal number of participants acceptable for a good psychometric analysis; the final sample comprised 128 participants. The Cronbach's alpha coefficient was used to determine internal consistency, which is considered good when the value is > 0.80 and most inter-item correlations are in the recommended range of moderate magnitude (0.15–0.50) (20).

#### CFA

We used the final sample (with 128 participants) to elucidate whether the CAPS-5 in the Brazilian context should have the same structure as the original CAPS-5, validated in the American context. The factor structure of the adapted CAPS-5 was examined using CFA. Items were treated as ordinal variables, and parameters were estimated using the maximum-likelihood estimator method, which provides good performance for small samples and a robust chi-square. The model fit was evaluated using chi-square under the degree of freedom ratio (X2/df): values < 3 are considered acceptable for the model; Goodness of Fit Index (GFI), Tucker-Lewis Index, Comparative Fit Index (CFI): values < 0.90 indicate a lack of fit, values between 0.90 and 0.95 indicate a reasonable fit, and values between 0.95 and 1.00 indicate a good fit; Root Mean Square Error of Approximation (RMSEA): values ≤ 0.06 indicate a close fit; and Standardized Root Mean Square Residual (SRMR): values < 0.08 indicate a well-fitting model (21, 22).

For comparative analysis, we performed the chi-square, One-Way ANOVA and Kruskal-Wallis for categorical and continuous variables. Demographic information was missing for up to six patients, depending on the variable, in Sample 2. Otherwise, no other missing data were detected. The significance level of the tests was fixed at 0.05. All statistical analysis were performed using SPSS version 21 (IBM Corporation, Armonk, NY, USA). The SPSS AMOS was used to perform CFA.

## Results

The sample included 128 patients: 97 patients from PROVE (Sample 1 and Sample 2) and 31 patients from HCPA-UFRGS (Sample 3). Most of the patients were female (93.8%), single (63.5%), employed (69.9%), and religious (66.9%). CAPS-5 total severity score and total amount of symptoms did not differ among samples (Table 1).

### Inter-rater Reliability

To estimate the inter-rater reliability, we considered the 32 individuals from Samples 1 and 2 who had CAPS-5 performed by two interviewers. The inter-rater reliability of the total PTSD symptom severity score was high [intraclass correlation coefficient = 0.994, 95% CI (0.987–0.997), *p* < 0.001]. Cohen's kappa for each item was evaluated to determine if there was agreement between the two raters. Among the 20 items from the CAPS-5 scale, we found total agreement in four (B5/Physiological distress; C1/Memory avoidance; E1/Irritability; E2/Recklessness). We found Kappa between 0.759 and 0.8 in five items (D2/Distressing dreams, *k* = 0.792; C2/External avoidance, *k* = 0.795; D2/Negative beliefs, *k* = 0.768; D4/Negative states, *k* = 0.759; E5/Concentration problems, *k* = 0.770). The remaining 15 symptoms resulted in a kappa value > 0.8, indicating an “almost perfect” agreement between raters (Table 2).

**Table 2:** Inter-raterreliability coefficients of CAPS-5 (*n* = 36).

**Factor**	**Item**	**Kappa**
B Intrusion	B1 Recurrent memories	0.880^*^
	B2 Distressing dreams	0.792^*^
	B3 Dissociative reactions	0.953^*^
	B4 Psychological distress	0.897^*^
	B5 Physiological reactions	1^*^
C Avoidance	C1 Memory avoidance	1^*^
	C2 External avoidance	0.795^*^
D Negative cognitions	D1 Dissociative amnesia	0.842^*^
	D2 Negative beliefs	0.768^*^
	D3 Distorted cognitions	0.960^*^
	D4 Negative states	0.759^*^
	D5 Diminished interest	0.813^*^
	D6 Feelings of detachment	0.952^*^
	D7 Reduction of positive emotions	0.956^*^
E Hyperarousal	E1 Irritability	1^*^
	E2 Recklessness	1^*^
	E3 Hypervigilance	0.857^*^
	E4 Startle response	0.871^*^
	E5 Concentration problems	0.770^*^
	E6 Sleep disturbance	0.823^*^

* *p < 0.001*.

### Internal Consistency

Cronbach's alpha coefficients indicated high internal consistency for the CAPS-5 full scale (α = 0.826) and an acceptable level of internal consistency (23) for the four symptom clusters: B/Intrusion (α = 0.631), C/Avoidance (α = 0.404), D/Negative cognitions (α = 0.701), and E/Hyperarousal (α = 0.537). Two symptoms, D1/Dissociative amnesia and E2/Recklessness, had low item-total correlations (0.025 and 0.095, respectively). The range of item-total correlations for the remaining 18 symptoms was 0.317–0.613, with a mean of 0.438. By excluding these two items, the Cronbach's alpha coefficient for the full scale increased to 0.842.

Most inter-item correlations were in the recommended range of 0.15–0.50 (20), with a mean of 0.193 across all 20 symptoms. The symptoms D1/Dissociative amnesia and E2/Recklessness exhibited low inter-item correlations, with values of 0.152 and 0.189, respectively. The mean inter-item correlation for the remaining 18 symptoms was 0.233.

### CFA

CFA with the maximum-likelihood estimation method was conducted to determine whether the factor structure indicated by the original scale could be confirmed. We performed CFA for the 20-item original CAPS-5 scale and for the 18-item model with the exclusion of two items (D1 and E2), suggested by the internal consistency analysis. The fit indices for the 18-item model were X2/gl = 1.441, GFI = 0.861, CFI = 0.878, TFI = 0.855, RMSEA = 0.059, and SRMR = 0.076, supporting a reasonable fit to the data. We concluded that the 20-item and 18-item CAPS-5 models did not fit the data adequately well.

In order to improve the performance of the instrument, we analyzed all factor loads from each item from the 18-item model. We found three items with low factor loads: D3/Distorted cognitions (0.388), E1/Irritability (0.305), and E3/Hypervigilance (0.400). All other items had factor loads > 0.40. We then performed a third CFA of the 15 remaining items. The 15-item model exhibited a good fit to the data (X2/gl = 1.248, GFI = 0.910, CFI = 0.948, TFI = 0.951, RMSEA = 0.044, and SRMR = 0.063) (Table 3).

**Table 3:** CFA fit statiscs for 20-item, 18-item, and 15-item CAPS-5 models.

**Fit index**	**Level of good fit**^**a**^	**20-item**	**18-item**	**15-item**
X2/df	<3	1.350	1.441	1.248
GFI	>0.9	0.854	0.861	0.910
CFI	>0.9	0.878	0.878	0.948
TFI	>0.9	0.858	0.855	0.951
RMSEA (90% CI)	<0.06	0.052 (0.033–0.069)	0.059 (0.039–0.077)	0.044 (0.000–0.069)
SRMR	<0.08	0.076	0.076	0.063

a *Brown (21) and Hu and Bentler (22)*.

On observing the final 15-item model, all items were found to have significant loadings onto their respective latent constructs. The standardized regression weights (factor loadings) for all items were >0.3, corresponding to good-magnitude loadings (21). The occurrence of factors with item reduction (two items in factor D/Negative cognitions and three items in factor E/Hyperarousal) may explain the relatively poor loadings. The item D5/Diminished interest exhibited a high factor load: 81% of the factor variance was accounted by this item, suggesting that D5/Diminished interest is a strong indicator of negative cognition. All other factor loadings ranged between 0.40 and 0.65 (Table 4).

**Table 4:** Parameter estimates for the 15-item CAPS-5 model.

**Factor**	**Item**	**Estimate**	**SE**	**FL**
B Intrusion	B1	1.00		0.45
	B2	1.88	0.47^*^	0.52
	B3	1.99	0.51^*^	0.50
	B4	1.04	0.26^*^	0.42
	B5	2.13	0.49^*^	0.62
C Avoidance	C1	1.00		0.48
	C2	1.41	0.36^*^	0.54
D Negative cognitions	D2	1.00		0.65
	D4	.52	0.12^*^	0.45
	D5	1.26	0.18^*^	0.81
	D6	.94	0.16^*^	0.60
	D7	1.03	0.19^*^	0.58
E Hyperarousal	E4	1.00		0.40
	E5	1.01	0.25^*^	0.41
	E6	1.12	0.26^*^	0.47

* *p < 0.001*.

*Estimate, unstandardized regression weights; SE, standard error of the unstandardized regression weights; FL, factor loadings (standardized regression weights)*.

We calculated the new Cronbach's alpha coefficients for the 15-item model and found improvement compared with the full scale. The 15-item model exhibited high internal consistency (α = 0.835), and the two factors with item reduction maintained acceptable levels of internal consistency: D/Negative cognitions (α = 0.747) and E/Hyperarousal (α = 0.403).

## Discussion

The present study describes the development of the Brazilian-Portuguese version of the CAPS-5 scale, a unique instrument for clinicians to evaluate PTSD in a structured manner in Brazil. Our research team has already translated the original English scale using a structured method published elsewhere (14). The present study now demonstrates a high inter-rater reliability for all 20 items and the total severity score. It is important to emphasize that the raters were experienced professionals in attending PTSD patients. Further studies with less experienced health-care professionals are necessary to determine the consistency of our results.

The present study has demonstrated an adequate internal consistency for the four clusters of symptoms and high internal consistency for the full scale. These findings are in line with previous scale validations for other languages (8, 9) as well as the validation of the original CAPS-5 version (7). Lower Cronbach's alpha coefficients for the cluster C/Avoidance have been reported before and can be explained by the existence of only two items in this cluster; similar results were found in the original English version and in the Dutch translated version (7, 9). On observing the items separately, we found that two items had a low item-total correlation (D1/Dissociative amnesia and E2/Recklessness), consistent with the findings of the original English version. According to the original CAPS-5 validation process (7), these findings may be attributable to a very infrequent endorsement of these two symptoms, corroborating the hypothesis that these items may be important but relatively rare symptoms of PTSD, or it may be that they are simply not representative of the PTSD construct.

A CFA was conducted to verify adequate fit to the data. Due to the similar validation process of CAPS-5 in previous studies and the existence of consolidated constructs (clusters) that explain PTSD, we decided to perform only a CFA instead of evaluating constructs from the entire scale (with exploratory factor analysis). Previous results from internal consistency analysis were used to suggest items for exclusion; the 15-item model provided the best fit to the data, compared to the full scale and 18-item model. We postulated that a 15-item model for CAPS-5 could maintain adequate results without compromising diagnostic capacity.

The use of heterogeneous data from different sources is a strength of the present study and is in contrast to the validation of the original scale, which was based on symptoms observed in only war veterans. It would be of interest for future studies to perform a more complete evaluation of the scale construct. A challenge observed in this study was the maintenance of the original structure and constructs/factors in the CFA: the occurrence of factors composed of few items can explain the relatively low factor loads. Other strong points are the well-trained professionals that were able to diagnose PTSD properly, only one research team undertaking the entire adaptation process of the scale, and strong inclusion criteria for the participants.

This study presents a limitation regarding the validation process. The main objective of this study was to adapt the scale for the Portuguese language and test its reliability and consistent validity. During the validation process, this study considered maintaining the original scale and its content validity; we did not intend to construct a different scale structure for the Brazilian context with the present results and to perform a complete validation study.

Another important limitation must be considered: although we were able to evaluate individuals from different institutions and sources, a significant number of participants were women who experienced a traumatic event related to sexual violence. To minimize the impact of using a convenience sample, we included participants from two different trauma centers and also 3 different samples, two of them with spontaneous demand, but it was observed that even in centers that receive all types of trauma in a naturalistic setting, a large number of patients are female who seek treatment for sexual assault. This is very common in a low- to middle-income country such as Brazil, where this type of trauma has a high prevalence due to the social context (24). However, this limitation compromises the generalization capacity of the study results. It is also important to mention that the original validation study for the English version had presented the same limitation, using a convenience sample composed only by war veterans, majoritarian male participants. Therefore, future studies should assess the consistency of results by comparing the 15-item scale with the 20-item scale for different types of traumatic events.

## Conclusion

In developing countries, PTSD is mostly related to urban violence that has a high frequency of traumatic events, such as robbery, kidnapping, sexual assault, rape, witnessing shootings, and other life-threatening situations. This is frequently related to complex PTSD diagnoses; thus, we must ensure that the CAPS-5 is an efficient instrument to detect this reality. Establishing a validated version of a Brazilian-Portuguese diagnostic instrument to evaluate PTSD symptomatology is extremely important for researchers to better understand these issues in this socio-cultural context.

## Data Availability Statement

The original contributions presented in the study are included in the article/supplementary material, further inquiries can be directed to the corresponding author/s.

## Ethics Statement

The studies involving human participants were reviewed and approved by Ethical Committee of UNIFESP and UFRGS. The patients/participants provided their written informed consent to participate in this study.

## Author Contributions

CZ and BC were involved with the data collection. VC had data analyzed. MMa reviewed the article and helped with the discussion and the data collection. LF, MMe, and AM guided all the research process and reviewed the manuscript. All authors contributed to the article and approved the submitted version.

## Conflict of Interest

The authors declare that the research was conducted in the absence of any commercial or financial relationships that could be construed as a potential conflict of interest.
